# Do we need to worry about eating wheat?

**DOI:** 10.1111/nbu.12186

**Published:** 2016-02-16

**Authors:** P. R. Shewry, S. J. Hey

**Affiliations:** ^1^ Rothamsted ResearchHertfordshireUK; ^2^ University of ReadingBerkshireUK

**Keywords:** coeliac disease, FODMAPs, food allergy, gluten, intolerance, wheat

## Abstract

Wheat is a staple food throughout the temperate world and an important source of nutrients for many millions of people. However, the last few years have seen increasing concerns about adverse effects of wheat on health, particularly in North America and Europe, with the increasing adoption of wheat‐free or gluten‐free diets. This relates to two concerns: that wheat products are disproportionally responsible for increases in obesity and type 2 diabetes and that wheat gluten proteins cause a range of adverse reactions, including allergies, coeliac disease and ‘non‐coeliac gluten sensitivity’. The first concern has been refuted in previous publications, and we therefore focus on the second here. Current evidence indicates that allergy to ingested wheat and coeliac disease (and related intolerances) each occur in up to 1% of the population. The extent to which their prevalence has increased is difficult to quantify due to improved diagnosis and increased awareness. However, neither appears to be increasing disproportionally when compared with other immunologically mediated adverse reactions to food. Other adverse reactions to wheat are more difficult to define as their mechanisms are not understood and they are therefore difficult to diagnose. In particular, ‘non‐coeliac wheat sensitivity’ has been reported to occur in 6% or more of the population in the US. However, the application of more rigorous diagnostic criteria is likely to give substantially lower estimates of prevalence. It is therefore unlikely that the health of more than a small proportion of the population will be improved by eliminating wheat or gluten from the diet. In fact, the opposite may occur as wheat is an important source of protein, B vitamins, minerals and bioactive components.

## Background

The last few years have seen increasing concerns, particularly in the media and lay press, about the effects of wheat‐based foods on health, with the increasing adoption of wheat‐free or gluten‐free diets. These concerns have largely been propagated through the media, particularly the popular press, Internet and social media, rather than conventional medical and public health channels, and the evidence base is often obscure. Nevertheless, the impact has been dramatic and of concern not only for wheat producers and the food industry but also for public health due to the impact on the intake of components, which are conventionally consumed in wheat products, such as dietary fibre, B vitamins and minerals (Steer *et al*. 2008).

The concerns can broadly be divided into two types: that wheat products are disproportionally responsible for increases in obesity and type 2 diabetes and that wheat gluten proteins cause a range of adverse reactions, including allergies, coeliac disease and a range of less well‐defined conditions. The role of wheat products in the increasing levels of obesity and associated conditions was promoted by the best‐selling book *Wheat Belly: Lose the Wheat, Lose the Weight, and Find Your Path Back to Health* (Davis 2011), which led to a proliferation of publications on wheat‐free diets and recipes. The scientific flaws in the arguments have been discussed in the scientific literature (Jones 2012; Brouns *et al*. 2013) but these scientific publications have had little or no effect on the public perception that wheat is bad for you!

**Figure 1:**
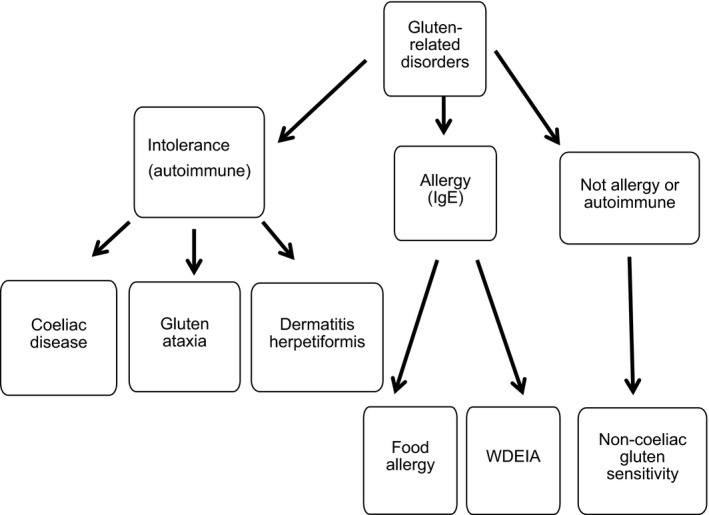
Nomenclature and classification of disorders proposed to be related to gluten in food. Modified from Sapone *et al*. (2012). Wheat‐dependent exercise‐induced anaphylaxis (WDEIA).

We therefore focus here on the second topic, considering the evidence for increases in adverse reactions to the consumption of wheat gluten (and possibly also other wheat components). The spectrum of conditions is summarised in Figure 1, which is based on the outcome of an international panel of experts which met in 2011 (Sapone *et al*. 2012). However, it should be borne in mind that this is a simplification and some conditions may occur together in the same patients. Finally, it is important to note that methods of diagnosis have improved greatly over the past few decades, together with increased awareness of food‐related conditions among clinicians and consumers. Hence, it is important to consider whether these changes have affected our estimates of prevalence.

## Wheat allergy

Allergies are hypersensitive responses to foreign components, most commonly proteins, and are usually associated with the production of a specific class of antibody called IgE (in contrast to the IgG antibodies which are produced in response to most invading pathogens). Symptoms of allergy to ingested wheat products include atopic dermatitis, urticaria (also called hives or nettle rash), and respiratory and gastrointestinal symptoms. A range of proteins has been implicated, notably α‐amylase inhibitors and gluten (gliadin and glutenin) proteins (reviewed by Tatham & Shewry 2008; Matsuo *et al*. 2015).

Zuidmeer *et al*. (2008) reported a detailed meta‐analysis of the prevalence of plant food allergies, which included data from 15 studies of wheat allergy. These comprised cohorts ranging between about 500 and 10 000 subjects with diagnosis by food challenge (but not always double‐blind), skin prick test and serum IgE analysis, and perception of wheat allergy measured by questionnaires. Four studies in which children (aged 3–14 years) were challenged with wheat in the diet showed a mean prevalence of 0.25% (7/2807) with a range from 0–0.5%. Two UK cohorts showed prevalences in children of 0.2% at 9–12 years and 0.3% at 6 years (Venter *et al*. 2006a, 2006b), but challenges of adults were not reported. Sensitisation to wheat, determined by reaction in a skin prick test, was higher in UK adolescents (15 years) (1.2%) (Pereira *et al*. 2005) than in UK children (4–11 years) (0.2–0.6%) (Arshad *et al*. 2001; Pereira *et al*. 2005; Venter *et al*. 2006a, 2006b) and a high level of sensitisation in adults (up to 3.6%) has also been reported for other countries based on the determination of IgE to wheat in the serum. It is often stated that wheat allergy is most common in infants and then disappears. This is consistent with the data on perception of wheat allergy, which tends to be higher than sensitisation or response to dietary challenge in children, but lower in adults. Of the 36 studies included in the meta‐analysis, only six used double‐blind food challenges and these showed that the prevalence of wheat allergy was within the ranges reported for allergies to other plant foods (fruit, nuts, vegetables, soya and sesame) (Zuidmeer *et al*. 2008).

## Wheat‐dependent exercise‐induced anaphylaxis (WDEIA)

The best characterised form of wheat allergy is wheat‐dependent exercise‐induced anaphylaxis (WDEIA). This is a type of allergic response, which is triggered by the ingestion of food followed by physical exercise, with wheat and crustaceans being the commonest causes (Beaudouin *et al*. 2006). WDEIA has been studied in most detail by Japanese workers, who recognise two forms (Yokooji *et al*. 2015). Conventional (CO) WDEIA is the dominant form and is considered to be sensitised via the gastrointestinal tract, with the major allergen being ω‐5 gliadin (Palosuo *et al*. 2001; Morita *et al*. 2003). However, a second form has also been defined in Japan, which appears to be sensitised via the skin and/or mucosa by hydrolysed wheat protein (HWP) present in soap. The major sensitising agent in HWP‐WDEIA appears to be γ‐gliadin and reactions can occur after exposure to soap or consumption of wheat (Yokooji *et al*. 2013).

The prevalence of food‐dependent exercise‐induced anaphylaxis (including WDEIA) has been reported as 0.017% in Japanese children (Aihara *et al*. 2001), while screening of 935 Japanese adults for wheat allergy (also including WDEIA) using questionnaires, skin prick tests and determination of ω‐5 gliadin‐specific IgE identified only two allergic subjects (0.21%) (Morita *et al*. 2012).

## Coeliac disease

Coeliac disease (CD) is an autoimmune condition, which affects the small intestine, resulting in malabsorption, weight loss, fatigue, abdominal pain, vomiting and diarrhoea. Consequently, patients with CD suffer from nutrient deficiencies including iron anaemia and folate deficiency. However, individuals may also be asymptomatic or present only mild symptoms.

The role of wheat gluten proteins in triggering CD is well‐established, with gliadin and glutenin proteins being the major cause (reviewed by Gilissen *et al*. 2014). Currently, 31 short peptide sequences in wheat gluten proteins, and related proteins in barley and rye, have been defined as being coeliac toxic: these are often referred to as coeliac ‘epitopes’. However, mapping is incomplete and the number of distinct epitopes is a matter of on‐going discussion (Sollid *et al*. 2012).

Although CD was historically considered as a paediatric condition, it is now recognised that it can present at any age, and large‐scale screening has revealed a substantial level of undiagnosed CD in adults. For example, a study of 7550 participants carried out in Cambridge (UK) showed that 1.2% of adults aged 45–76 years were serologically positive (West *et al*. 2003). Similarly, analysis of 16 847 adults aged 50 years or more in Minnesota showed 0.8% undiagnosed CD (Godfrey *et al*. 2010). Hence, the prevalence of CD in Europe and countries with high proportions of populations of European ancestry (*e.g*. the US, Australia) is now widely estimated as about 1% of the population, although substantial variation occurs between countries, from as low as 0.2% to over 5%. Within Europe, Finland has a particularly high incidence, reported as 1–2.4% (Maki *et al*. 2003; Godfrey *et al*. 2010; Mustalahti *et al*. 2010; Walker *et al*. 2010; Rubio‐Tapia *et al*. 2012).

There is a perception that the prevalence of CD is increasing, although this may be the result, at least in part, of increased awareness and improved diagnosis (with screening for the presence of antibodies to the enzyme tissue transglutaminase in the serum being used for initial diagnosis) (Ludvigsson *et al*. 2015). An increased prevalence in Sweden has been attributed to changes in infant feeding (Olsson *et al*. 2008; Myléus *et al*. 2009) while Lohi *et al*. (2007) reported a two‐fold increase in CD in Finnish adults between 1978–1980 and 2000–2001 (from 1.05% to 1.99%), after adjusting the data for improved diagnosis over the same period.

Green and Cellier (2007) note that adult CD is about twice as prevalent in women as in men, in common with higher prevalences of other autoimmune diseases. However, they also note that women are more likely to suffer from iron deficiency anaemia and osteoporosis, symptoms which may lead to investigations by health professionals. The prevalence in women has also been reported to decrease after about age 65 years (Green *et al*. 2001).

Even when the initial serological screening is confirmed by small bowel biopsy, patients may not experience changes in bodyweight or other symptoms. Nevertheless, the association of CD with increased risk of a range of other disorders (Corrao *et al*. 2001; Green *et al*. 2003; West *et al*. 2004; Green & Cellier 2007; Solaymani‐Dodaran *et al*. 2007; Godfrey *et al*. 2010) means that treatment is required even in the absence of symptoms.

## Conditions related to coeliac disease

Coeliac disease may be associated with neurological conditions, with peripheral neuropathy and gluten ataxia (GA), in which the cerebellum is damaged, being the most common. Their prevalence has not been established but Hadjivassiliou *et al*. (2002) have estimated that neurological dysfunction may occur in about 6–10% of patients presenting with gastrointestinal symptoms. These authors reviewed 35 publications in which ataxia and peripheral neuropathy were each present in 29 of 83 patients. However, similar symptoms are also observed in patients defined as suffering from ‘gluten sensitivity’, in the absence of diagnosed CD (Hadjivassiliou *et al*. 2010). More recently, Hadjivassiliou *et al*. (2015) have reported that GA has a prevalence of 15% among all ataxias.

Dermatitis herpetiformis (DH) is a form of CD, which presents as a chronic skin disease. Its prevalence is much lower than typical CD, estimated at 0.001– 0.04%, and in common with CD, it is higher in populations of European descent and low in Asian and African‐American populations (Gawkrodger *et al*. 1984; Mobacken *et al*. 1984; Smith *et al*. 1992; Bolotin & Petronic‐Rosic 2011). In contrast to CD, the prevalence of DH is from 1.5–2 times higher in men than in women (Smith *et al*. 1992)

## Schizophrenia and autism spectrum disorder

Associations between the consumption of wheat and milk and schizophrenia and autism spectrum disorder have been studied in some detail, with both conditions being improved in some patients by interventions with gluten‐free, casein‐free or gluten and casein‐free diets (Singh & Roy 1975; Christison & Ivany 2006; Kalaydiian *et al*. 2006; Whiteley *et al*. 2010, 2013).

It has been hypothesised that neuroactive peptides released by digestion of wheat gluten are responsible for neurological effects (Dohan 1979; Dohan *et al*. 1984), which has given rise to the concept of gluteomorphins. These are proposed to be opioid peptides that are released by digestion of gluten in the gastrointestinal tract and taken up into the bloodstream, resulting in neurological effects and ‘addictive’ properties. Similarly, casomorphins have been suggested to be responsible for similar symptoms associated with milk consumption. However, there is little experimental evidence for this hypothesis.

## Non‐coeliac gluten sensitivity (NCGS)

In recent years, an increasing number of patients have reported symptoms related to wheat consumption, which are not classical allergic or autoimmune responses. This has led to the definition of a new condition called ‘non‐coeliac gluten sensitivity’ (NCGS) (Sapone *et al*. 2012). The range of symptoms varies widely, including gastrointestinal symptoms, tiredness, headache, dermatitis, pains in muscles and joints, depression, anxiety and anaemia, and it is not clear whether NCGS represents a single syndrome or a range of conditions (Sapone *et al*. 2012). It is therefore best defined in negative terms: as a reaction to gluten (or wheat) when both CD and allergy have been excluded (Aziz *et al*. 2012; Sapone *et al*. 2012). Furthermore, the role of gluten has not been clearly established and the symptoms could relate to other grain components. Hence, the term ‘non‐coeliac wheat sensitivity’ (NCWS) may be more appropriate (Carroccio *et al*. 2014; Catassi *et al*. 2015).

The pathogenesis of NCGS/NCWS is not understood but is likely to feature a mixture of factors including the stimulation of the innate immune system. This lack of understanding poses a challenge for diagnosis but the recent report of an expert group recommends a gluten‐free diet followed by a double‐blind, placebo‐controlled gluten challenge, with variation of 30% or more in one to three main symptoms being a positive result in both phases (Catassi *et al*. 2015).

The true prevalence of NCGS/NCWS will not be clear until these criteria are rigorously applied. The prevalences reported in previously published studies are therefore likely to be higher than the true values. For example, NCGS was diagnosed in 6% of 5896 patients seen at the Center for Celiac Research in Maryland, USA (Sapone *et al*. 2012) while Volta *et al*. (2014) identified 3% of 12 000 patients with suspected NCGS in a multisite study of outpatients in Italian health centres. A survey of 1002 adults in the Sheffield area of the UK identified 13% with self‐reported gluten sensitivity (GS) while a further study of 200 GS patients showed that 7% had CD and 93% NCGS (Aziz *et al*. 2014). The ratio of females to males in the latter two studies was about 4:1 (Aziz *et al*. 2014; Volta *et al*. 2014).

## FODMAPs and gastrointestinal disorders

Wheat, in common with other plant foods, contains small fermentable carbohydrates, which have been termed FODMAPs (Fermentable, Oligo‐, Di‐, Mono‐saccharides And Polyols). The most abundant of these are fructo‐oligosaccharides (fructans) (up to about 2% dry weight), sucrose (0.5‐1.5% dry weight) and raffinose (0.2–0.7% dry weight) (reviewed by Shewry & Hey 2015). A low FODMAP diet may improve the management of irritable bowel disease (IBS) and inflammatory bowel disease (Crohn's disease and ulcerative colitis), by reducing fermentation in the colon (Gibson & Shepherd 2010; Staudacher *et al*. 2011; Muir & Gibson 2013). Furthermore, a tightly controlled intervention trial with self‐reported NCGS patients showed a significant improvement of symptoms during a low FODMAP run‐in period, but no effect of gluten challenge (Biesiekierski *et al*. 2013). The low contents of FODMAPs in gluten‐free products may therefore account for improvements experienced by IBS and NCGS patients on gluten‐free diets (Muir & Gibson 2013; Biesiekierski & Iven 2015).

## Wider impact of gluten‐free diets on nutrition and health

Concern among the general public about the impact of wheat on health is increasing dramatically, particularly in the US where a third of adults have stated their wish to cut down or eliminate gluten consumption. This has created a valuable market for gluten‐free foods, estimated as worth $1.77 billion in the US ($3.42 billion globally) in 2013 and forecast to increase to almost $24 billion in the US by 2020 (Statista 2015). The numbers who are consuming gluten‐free diets now greatly exceed even the highest estimates for the prevalence of gluten‐related effects on health, which raises the question of how the changes in diet are affecting the dietary intake of nutrients and bioactive compounds. In the heat of the debate about the adverse effects of gluten, it is often forgotten that wheat, and other cereals, make a much broader contribution to diets. For example, the UK *National Diet and Nutrition Survey* (*NDNS*) showed that bread alone contributes 11% of the daily intake of protein, 18–21% of dietary fibre (non‐starch polysaccharides), 15–16% of thiamine (vitamin B_1_), 10–11% of niacin (vitamin B_3_), 12% of folates (vitamin B_9_), 15–16% of iron, 15–19% of calcium, and substantial proportions of a number of other essential micronutrients to the diets of UK adults (Bates *et al*. 2014). In addition, wheat (particularly wheat bran) is rich in a range of phytochemicals, including phenolic acids and betaine, which may have health benefits (reviewed by Shewry & Hey 2015). Several studies have shown that gluten‐free foods may be depleted in protein and micronutrients compared to conventional diets (Thompson 1999, 2000; Kinsey *et al*. 2008; Pellegrini & Agostini 2015; Wu *et al*. 2015) and food scientists have identified the challenge of improving the nutritional quality and health benefits of gluten‐free breads (Capriles *et al*. 2016).

## Conclusions

Whereas adverse reactions to wheat could be considered to be well understood only a decade ago, the landscape has since become immensely more complicated. Wheat allergy remains the best understood condition, and the most readily diagnosed. The prevalence appears to be below 1% with WDEIA (which can result in anaphylaxis) being much rarer. There is no evidence that the prevalence is increasing disproportionally compared with other food allergies or that the prevalence is related to the types of wheat or wheat products that are consumed. The current prevalence of CD in the UK is also probably about 1%, but it is not clear whether the increases that have been observed in many countries reflect true increases in prevalence or result from greater awareness and improved diagnosis.

Other conditions related to wheat gluten, or other components of the wheat grain, are less well understood and diagnosis is problematic. However, there is no doubt that the prevalence is much lower than the proportions of consumers in North America and Western Europe who prefer gluten‐free diets, or the numbers who self‐report for NCGS, perhaps of the same order as allergies and CD. The agreement of diagnostic criteria for NCGS is therefore an important step for determining true prevalence while controlled interventions are also needed to identify whether wheat gluten, FODMAPs or other grain components are responsible.

It is therefore an over‐reaction to assume that the health of more than a small proportion of the population will be improved by eliminating wheat or gluten from the diet. In fact, the opposite may occur as wheat is an important source of protein, B vitamins, minerals and bioactive components.

Finally, it is important to note that wheat is the major staple food in much of the temperate world, including developing countries in North Africa and West and Central Asia, where it may contribute between 50–70% of total food intake, and parts of China and India. It is also contributing increasingly to the diet in Sub‐Saharan Africa. Although data are limited, there is little evidence that these countries are experiencing increases in adverse reactions to wheat consumption, and decreased wheat production due to concerns emanating from prosperous western countries would have a disastrous impact on food security.

## Conflict of interest

The authors have no conflict of interest to disclose.
